# Standardization of Broadband UV Measurements for 365 nm LED Sources

**DOI:** 10.6028/jres.117.004

**Published:** 2012-02-02

**Authors:** George P. Eppeldauer

**Affiliations:** National Institute of Standards and Technology, Gaithersburg, MD 20899, USA

**Keywords:** 365 nm LED, broadband UV measurements, Hg source, standard UV measurement, ultraviolet irradiance, UV excitation source, CIE UV-A meter

## Abstract

Broadband UV measurements are evaluated when UV-A irradiance meters measure optical radiation from 365 nm UV sources. The CIE standardized rectangular-shape UV-A function can be realized only with large spectral mismatch errors. The spectral power-distribution of the 365 nm excitation source is not standardized. Accordingly, the readings made with different types of UV meters, even if they measure the same UV source, can be very different. Available UV detectors and UV meters were measured and evaluated for spectral responsivity. The spectral product of the source-distribution and the meter’s spectral-responsivity were calculated for different combinations to estimate broad-band signal-measurement errors. Standardization of both the UV source-distribution and the meter spectral-responsivity is recommended here to perform uniform broad-band measurements with low uncertainty. It is shown what spectral responsivity function(s) is needed for new and existing UV irradiance meters to perform low-uncertainty broadband 365 nm measurements.

## 1. Introduction

**Fig. 1:**
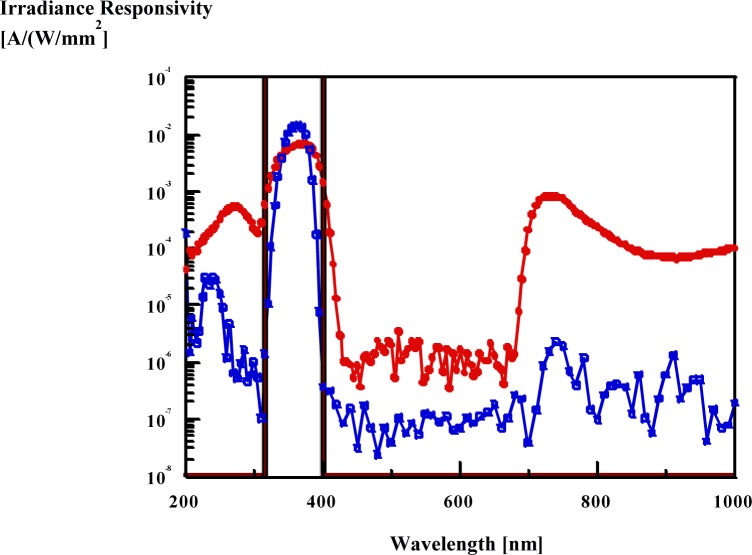
The CIE standardized UV-A (square-shape) and two realized spectral responsivity functions.

UV irradiance meters measure optical radiation from broad-band UV sources that peak at 365 nm for performing non-destructive testing of metal parts. Available UV-A irradiance meters read different irradiance values when they measure the same 365 nm source. The differences in the readings can increase to higher than 20 % when different UV radiometer models are involved in the irradiance measurements. The reason for the large measurement errors is lack of a proper standard spectral-responsivity function for the UV meters. The CIE standardized UV-A function has a square shape between 320 nm and 400 nm. Since filter combinations are to be used to realize this square-shape band-pass function, the spectral mismatch errors are large. The function-realizations of two commercial UV-A meters are illustrated in Fig. 1 [1].

The full dots show a typical realization and the open-squares represent a better realization. It is shown clearly that both realizations have large spectral mismatch errors relative to the CIE (square shape) standard function. Table 1 shows the measurement errors obtained with the better realization when four types of (commonly used) calibration sources are reused as test sources [1]. When the test source is different than the calibration source, the measurement error can increase to 60 % or higher. The measurement errors, using the typical meter, can increase to 300 %.

**Fig. 2:**
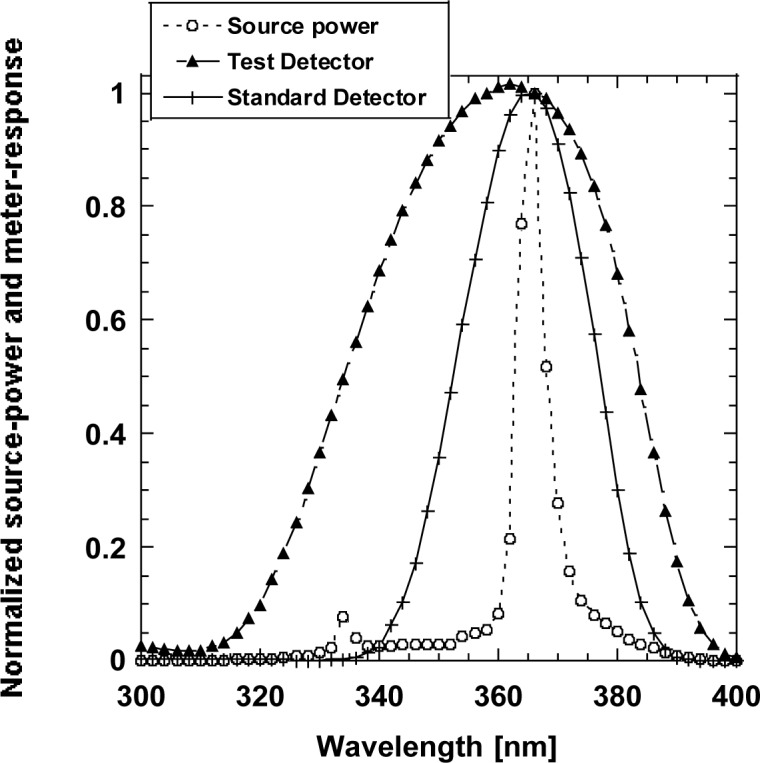
Relative spectral distribution of a filtered 365 nm Hg source and the responses of two different UV meters normalized at 366 nm.

A reasonable calibration (correction) factor for broadband UV measurements cannot be assigned based on the CIE UV-A standard responsivity function. Also, the spectral power distribution of the excitation 365-nm sources has not been standardized which is a must if the realized spectral responsivity function of the UV meter is different from the standard function. The wavelength range of the presently used CIE standard function is too broad for the required tests where 365 nm excitation sources are used. At present, the 365 nm Hg line is used for non-destructive material tests. The neighboring Hg-lines (such as the 334 nm line) are attenuated using glass filters. As shown in Fig. 2, the blocking is usually not perfect and the UV irradiance meters with their different spectral responsivity functions can measure the radiation produced by the remaining neighboring line(s). Also, the continuum of the source spectral distribution can produce different output signal components in the different broadband UV irradiance meters.

In this example, the test detector measured 14 % higher than the standard detector. The reason of this large error is that the spectral responsivities of the UV meters are different. At present, manufacturers are unable to improve their UV meters because of the lack of standardization of broad-band UV measurements. The UV sources have not been standardized either.

In order to phase out mercury from future tests (primarily because of safety reasons) and to eliminate out-of-band radiation from the calibration source, LED sources with similar irradiance distribution to the filtered Hg-lamps are introduced here.

The goal of this work is to make the broadband UV measurements uniform and to lower the broadband UV (with a typical emissivity peak of 365 nm) irradiance measurement uncertainties. To achieve this goal, the realization issues of the meter’s spectral responsivity and the 365-nm source distributions have been studied and a new definition for standardized measurements is developed to decrease the large errors in existing broadband UV measurements.

## 2. UV Detectors

**Fig. 3:**
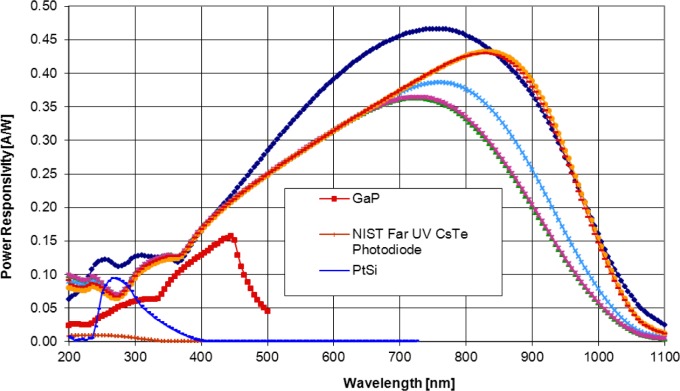
Spectral power responsivities of UV detectors. The broadband detectors (without labels) are different Si photodiodes.

The spectral power responsivities of several commercially available UV detectors have been measured. The obtained responsivity functions are shown in Fig. 3. The GaP detector, that has a reasonably high responsivity at 365 nm, cuts down at wavelengths longer than 500 nm. However, it is not resistant to UV damage. The PtSi detector cuts down for wavelengths longer than 400 nm. The CsTe photodiode also cuts down above 400 nm but its peak responsivity is almost a decade lower. The broadband detectors (not labeled) are mostly UV damage-resistant silicon photodiodes. Care should be taken when using these Si detectors because they measure optical radiation to about 1200 nm.

## 3. UV Irradiance Meters

**Fig. 4:**
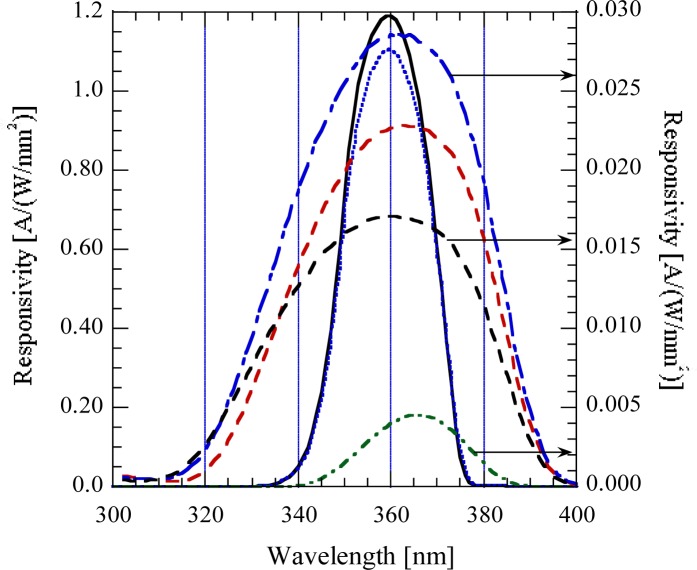
Spectral irradiance responsivities of different UV meters.

Six available UV irradiance meters have been measured for spectral irradiance responsivity. Figure 4 shows how different the spectral responsivities are. Both the peak responses and the spectral bandwidths (wavelength coverage) are different.

**Fig. 5:**
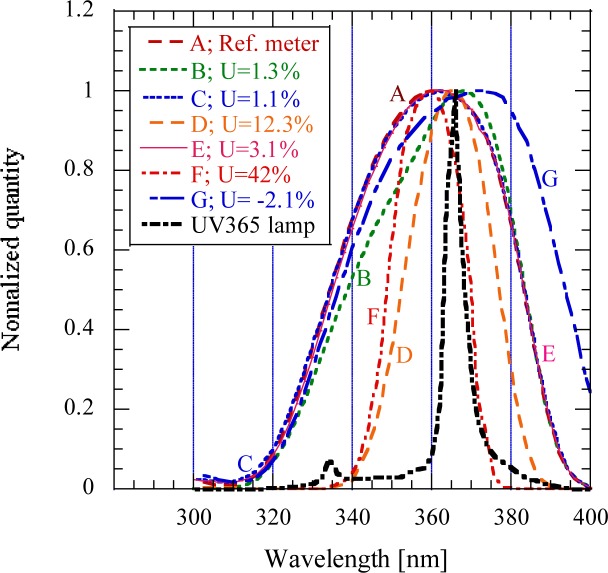
Normalized quantity versus wavelength includes the spectral responses of UV meters from A to G and the spectral power distribution of a filtered UV 365 nm Hg lamp.

Seven normalized spectral responses are shown in Fig. 5. The graph also shows the spectral distribution of the filtered Hg source. In order to determine the measurement errors of the different meters, the signal reading of each meter was divided by the signal reading of the commercial reference meter A (UDT268UVA)^1^ when they all measured the same 365 nm source in the figure. The percent errors are shown in the legend of the graph. The meter A was arbitrarily selected here as a reference meter to compare the measurement results obtained with the different UV meters. The errors show how uniform the seven broadband UV measurements are. Some of the shown measurement uniformity errors are small but others (where the shapes of the spectral responsivity functions were not adequate) increased to 42.3 %. In this worst case, the responsivity peak was 360 nm instead of 365 nm where the source peak was. Two meters (with narrow spectral bandwidths) did not measure the 334 nm (filter-attenuated) neighbouring Hg line and also part of the source continuum but the others with the broad spectral coverage measured both the 334 nm line and the continuum of the Hg source.

## 4. Spectral Modeling

**Fig. 6:**
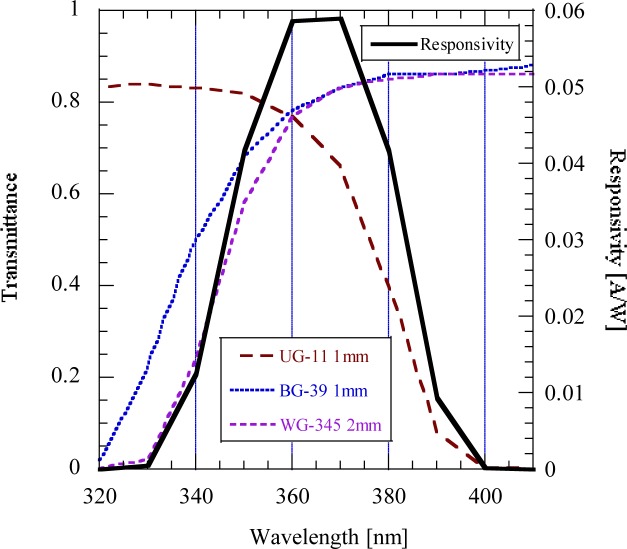
Modeled 365 nm response function using 3 filters and a UVG-100 silicon photodiode.

A spectral responsivity function that can be easily realized and used as a standard function has been designed using three filters and a UV-damage resistant silicon photodiode. The filter transmittance functions and the resultant responsivity function with its center at 365 nm are shown in Fig. 6. This design was made for LED sources with 365 nm +/− 5 nm peaks.

**Fig. 7:**
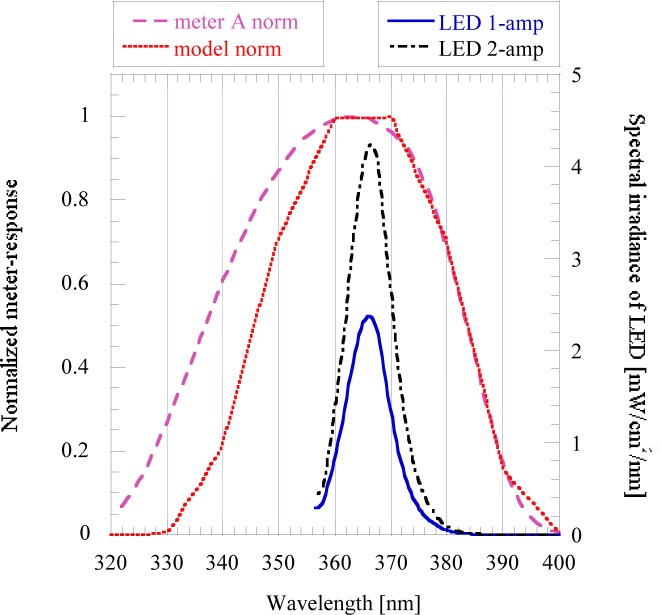
Comparison of the modeled 365 nm response to the commercial meter A. The distribution of the UV LED projector is shown at 1 A and 2 A currents.

The modeled and normalized response function is also shown in Fig. 7 together with the normalized spectral response of a commercial UV meter and the spectral distributions of a UV LED-based projector built with seven LEDs and dyes (phosphors) in an array. The meter responses in Fig. 7 were normalized to unity at the peaks to compare the two functions for 365 nm LED measurements.

It can be seen that the spectral power distributions of the two LED sources (shown for 1 A and 2 A feeding currents) are well within the spectral responsivity functions for both UV meters. Also, there is no leaking responsivity for wavelengths outside of the source spectral distribution function that could be measured by the two different meters. The requirement for standardization is to receive the same spectral product for the source distribution and the meter responsivity even if different meters or different 365 nm sources are used.

## 5. LED Sources

Because of environmental safety reasons, 365 nm LED sources have been selected to substitute Hg lamps. The spectral distribution of these LED sources is similar to that of Hg lamps. However, LED sources do not have neighboring emission lines and they do not have a continuum-radiation like Hg lamps. The first step in the modeling and in the following standardization is to establish the spectral band-limits and peak tolerances for the 365 nm LEDs. The suggestion here is to use LEDs with 365 nm +/- 5 nm peaks and a maximum spectrum halfwidth (FWHM) of less than 15 nm to keep the spectral mismatch errors and the uncertainties of the spectral products (signals) at a reasonably low level even if different meters and/or sources are used.

**Fig. 8:**
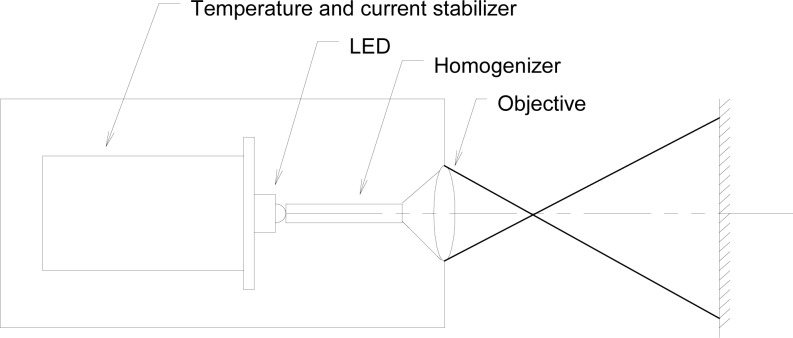
Input optics of a UV LED irradiance source

Figure 8 shows the design of a high-power UV-LED source that can produce a uniform irradiance, larger than 1 mW/cm^2^ [2] within a diameter of 7.5 cm at a distance of 40 cm (measured from the source). The spatial distribution of the radiance of the LED is not uniform. Therefore, a hexagonal quartz rod is used to homogenize the LED output radiation. The radiation incident into the rod, above an angle limit, is totally reflected inside of the rod-walls. The mixed reflected beams at the output of the rod will produce a uniform radiance distribution. The output beam of the homogenizer is focused by an objective lens onto the target surface. A 365 nm irradiance source like this can satisfy the requirements for standardization.

## 6. Definition of Uniform Broadband 365 nm Measurements

First, satisfy the requirements for source distribution: Use LEDs with 365 nm +/− 5 nm peaks and a maximum spectrum-half-width (FWHM) of less than 15 nm. In the next step, match the spectral response of the UV meters to the 365 nm source distribution function such that the spectral product of the source-distribution and the meter responsivity will produce an error less than the required measurement uncertainty when different UV meters (models) and/or different 365 nm sources are used.

The standardization of the broad-band UV measurements is needed only if more than one UV meter and/or more than one 365-nm source are used.

## 7. Conclusions

UV irradiance measurements verified that the presently applied CIE-A standard responsivity function is not suitable for accurate realization. Large 365 nm irradiance measurement uncertainties were obtained using the present CIE standard UV-A function. Up until now, Hg lamps were used for 365 nm excitation. The lamps were filtered but the spectral distribution of the excitation UV sources has not been standardized. Commercially available UV detectors and meters have been evaluated and compared in this paper. Also, different UV LED sources have been studied, evaluated, and suggested for standardization. A broadband UV measurement-model is described to develop an easily realizable spectral responsivity function based on the spectral power distribution of 365 nm LED sources. The 365 nm LED sources do not have environmental safety issues like mercury lamps and have continuum-free spectral distributions. Based on the model, a standard procedure is suggested that can result in uniform broadband 365 nm irradiance measurements with low uncertainty. The definition for the standard procedure is described. The first step in the procedure is to select the source according to the requirement described in the definition. In the second step, the responsivity function of the meter is selected with a spectral match to the standardized source-distribution function according to the definition. Invariant spectral products can be obtained when using the described 365-nm LED distributions and the meter responsivity functions even if different sources (with different peak wavelengths and spectral widths) are used in the measurements. Based on the definition described here, UV meter models (that satisfy the described standardization requirements) can be selected for uniform broadband 365 nm measurements. Other UV meters, where the described spectral-response requirements are not achieved, are non-ideal for uniform broadband UV measurements.

## Figures and Tables

**Table 1:** Errors in broad-band UV measurements with a commercial UV meter having a better-than-average response function.

Calibration Source		Test Source		
	FEL	Mercury	Deuterium	Xenon
FEL	0.0 %	50.8 %	−6.7 %	2.7 %
Mercury	−33.7 %	0.0 %	−38.2 %	−31.9 %
Deuterium	7.2 %	61.7 %	0.0 %	10.1 %
Xenon	−2.7 %	46.8 %	−9.2 %	0.0 %

## Footnotes

1 Certain commercial equipment, instruments, or materials are identified in this paper to foster understanding. Such identification does not imply recommendation or endorsement by the NIST, nor does imply that the equipment are necessarily the best available for the purpose.
